# Music groups and connectivity: Older adults' perceptions of socialising through community music

**DOI:** 10.1111/ajag.70057

**Published:** 2025-06-22

**Authors:** Helen English, Aimee Corderoy

**Affiliations:** ^1^ School of Humanities, Creative Industries & Social Sciences College of Human & Social Futures, University of Newcastle Newcastle New South Wales Australia

**Keywords:** aged, education, continuing, music, social environment, social support

## Abstract

**Objectives:**

While evidence for the benefits of engagement with music‐making as we age is well‐established, we know less about older adults' perceptions of and aspirations for involvement in music‐making. This article aimed to discover older adults' experiences of connectivity through and in music, and what enables positive experiences. It draws on a community music case study with older adults in Hobart, Tasmania, in 2023, one of five in a larger research project on creative ageing with music.

**Methods:**

The methodology was phenomenological, using a participatory approach to engage diverse participants in collaborative and culturally sensitive research that foregrounded the participant voice. Fifty‐five participants were recruited from five music groups: two choirs, a ukulele group, a string band and a four‐piece band. Methods were observation, participation, discussions and interviews. The analysis employed a reflexive thematic approach.

**Results:**

Four themes relating to connectivity were generated from engagement with the data: connecting with music; support to learn and realise musical selves; connections through music and beyond; and heightened connections and musical experiences.

**Conclusions:**

Participants stressed the importance of the welcome and accessibility they experienced in music groups to overcome self‐doubt and shared how important connections and social support were for their motivation and personal and musical growth.

**Policy impact**

This article contributes to a gap in our knowledge about what older adults want to learn, how they want to learn and what is fulfilling and meaningful for them. This will inform policy on promoting healthy and productive ageing.

## 1 INTRODUCTION

In this article, we present a case study within a larger study into creative ageing through music. The focus is on participants' experiences of connectedness through music, the perceived positive effects and what enables these. Therefore, as advocated for by Withnall,^1^ we foreground older adult music participants' voices through a participatory methodology and use reflexive thematic analysis.^2^

Maintaining and renewing social connections that may have been lost in late‐life transitions, such as retirement or widowhood, is critical to well‐being and identity.^3^ Participating in group music activities supports the mental health and well‐being of older community members by enhancing their sense of identity; affording them personal growth, with associated fulfilment; and fostering social connections.^4^, ^5^, ^6^, ^7^

In an exploratory study by English, Monk and Davidson (2018),^8^ music group members described musical learning environments in which acceptance and non‐judgement led to positive connections, and where people felt understood and supported despite personal challenges or different states of mind. Similar themes of connection are presented in studies where inclusivity and coming together were found to promote social well‐being and create perceived visibility for older adults.^9^, ^10^ Nyquist et al.^10^ ^(p. 110)^ recount how in group discussions, all members of three VocalEssence choirs (numbers not given) reported no longer feeling defined by health condition or age, but viewed themselves as ‘stronger, capable and more independent’, which also extended into improved social connections.

Building social connections and a strong sense of community increases social capital and encourages meaningful contributions to the broader community.^11^, ^12^ In a study of participants in the *Music for Life Project* in the UK, Varvarigou et al.^13^ find that older adults experience positive feelings through supporting less experienced members of choirs and instrumental groups and performing for the community. Dyer^14^ argues that the value of community ensembles extends beyond personal fulfilment, serving as an empowering platform for social justice and enabling members to advocate for shared causes and contribute to positive social change. The positive association with community is also reflected in Langston and Barrett's^12^ finding that the choir served as a powerful community resource facilitating trust, learning, social involvement and fellowship.

Gembris^15^ argues that musical capacities can be activated at any time in life. The opportunity to engage in music after retirement from work or caring can be experienced as a reconnection with early musical selves.^16^ This adoption of musical selves as a major later life interest is explored in the literature,^14^, ^17^ with themes of accomplishment, personal growth, lifelong learning and transfer of musical knowledge into everyday life highlighted when participants are supported to explore their musical selves within a safe and comfortable environment.^18^, ^19^ This is evident in a study of Circlesongs, a week‐long summer workshop in the Hudson River Valley, USA, in which Lehmberg^20^ details the psychological challenge of musicianship for the 26 older members who participated in her study. Participants stated that their connections to each other fostered a safe and trusting environment where they could challenge their vulnerability and self‐doubt, harnessing their musical selves and learning socially.

Reconnecting with or discovering musical identities through community music can be an aspirational journey that older adults previously suppressed or deemed unattainable.^17^ Research by Creech et al.^17^ offers valuable insights through the concept of ‘possible selves’, adapted from Markus and Nurius.^21^ This concept suggests that in early life, we envision versions of ourselves that shape our aspirations and actions; these imagined selves are fragile and can be obscured by negative feedback and/or experiences. By creating an inclusive environment and addressing internalised barriers, older adults can reconnect with their imagined musical selves.^17^ This can be appreciated in Dabback's^16^ study, where membership in New Horizons Music programs created an avenue to explore new paths, new goals and new identities.

Group music facilitates deep, non‐verbal communication that transcends words.^22^ When groups perform in settings such as nursing homes or participate in activist causes, their music can convey empathy and solidarity without the need for explicit language.^23^ Non‐verbal communication in music facilitates a unique form of empathic interaction that often surpasses verbal communication.^24^ The concept of non‐verbal communication in music involves a dynamic and fluid interaction of sounds, gestures, facial expressions, body movements, each carrying meaning in the absence of explicit verbal cues.^25^ Schiavio et al.^25^ describe how meaning through music is evolving and interactively negotiated in the moment through the continuous interaction and adjustment of participants to one another in a reciprocal process, without the need for explicit instruction. Environments that harness the participatory sense‐making that music offers can promote embodied and emotional communication, facilitating friendships and the formation of new communities.^26^ Through music, individuals create and participate in shared worlds of meaning.^26^

Research into lifelong learning in music has shifted from a focus on demonstrating there are well‐being benefits, as part of early advocacy for music's power, to a deeper engagement with older participants to understand what they are looking for, what they value in music‐making opportunities and their relationship to music itself on the ageing journey.^1^, ^27^ The aim of this case study was to hear from participants about their experiences, what was important to them, and what about their group supported them to learn and grow. To facilitate this, we used group discussions as the main mode of data collection.

## 2 METHODS

Foregrounding participants' voices calls for a qualitative approach and inductive analysis guided by the participants' reflections. The approach taken here was praxis‐based participatory research,^28^ which aims to engage diverse participants in a collaborative and culturally sensitive process, and allows them to be as involved as they wish. Groups for the case study were identified early in 2023 when Author 1 visited Tasmania. Organisations (music groups) were contacted by email and provided with organisation information statements and consent forms. On consenting, organisations distributed information and consent forms to potential participants. In mid‐2023, Author 1 travelled to Hobart to attend rehearsals and introduce the project, at which time recruitment was completed. Ethical approval was obtained from the University of Newcastle College Human Ethics Advisory Panel (H‐2022‐0252). Questions were open‐ended and can be found in Appendix S1. This study was approved by the University of Newcastle College Human Ethics Advisory Panel (H‐2022‐0252).

### 2.1 Data collection

Author 1 attended rehearsals, also participating, to get background and a feeling for each group. Data were collected through group discussions and interviews over a 15‐day period, which were audio‐recorded. Some music groups preferred whole group discussions during their rehearsal times (ukulele group, string band and four‐piece band) while the others opted for smaller group discussions outside rehearsal times (both choirs). Interviews (3) were used for people who could not make the discussion times. Participants were also invited to complete an anonymous online demographic survey. The richness of data, rather than a numerical sample size, guided completion of collection.^29^

### 2.2 Music groups and participants

There is a lively community music scene in Hobart, Tasmania, with a strong folk influence. While choirs and ukulele groups are the most accessible music groups in terms of prior learning and therefore tend to be well‐patronised, there are also niche groups, including an old‐time string band, a wind and string folk‐based group and rehearsals organised by the Folk Federation of Tasmania. Older adults (60 years and over) dominate membership as a demographic with time to spend on pursuing musical interests.

Fifty‐five members were recruited from five music groups (two choirs and three instrumental groups) that use a facilitator rather than a conductor–director and emphasise playing and learning together (Table 1). All groups are non‐auditioned, rehearse once a week and perform regularly. Participants range from 50 years old up, with the majority in their 70s and 80s, who were mainly born in Australia or England; one participant identified as Aboriginal (Table 1).

**TABLE 1:** Gender, age and place of birth of research participants.

Group	Female, *n*	Male, *n*	Average age years	Place of birth
Women's choir	12	0	68.5	England, Australia
Union choir	3	2	72	Australia, The Netherlands, England, Germany
Ukulele group	15	7	74	Australia, Australia (First Nations), England, USA
String band	6	7	72	Australia, England
Four‐piece band	1	2	No data	Australia
Totals	37	18	71.5 years	

### 2.3 Analysis

The analysis followed Braun and Clarke's reflexive thematic analysis approach^2^ in which, while having carefully considered questions prepared, the emphasis is on the co‐creation of meaning between interviewer and interviewee. After replacing participants' names with pseudonyms, chosen at random, both authors embarked on close reading and coding of the transcripts. This process produced tables and summary narratives for each music group. These were shared with the research participants for comment before preparing an early version of the findings for presentation at the 2024 Australian Association of Gerontology conference in Hobart, Tasmania, to which research participants were invited. Women's choir members attended and provided feedback.

**FIGURE 1:**
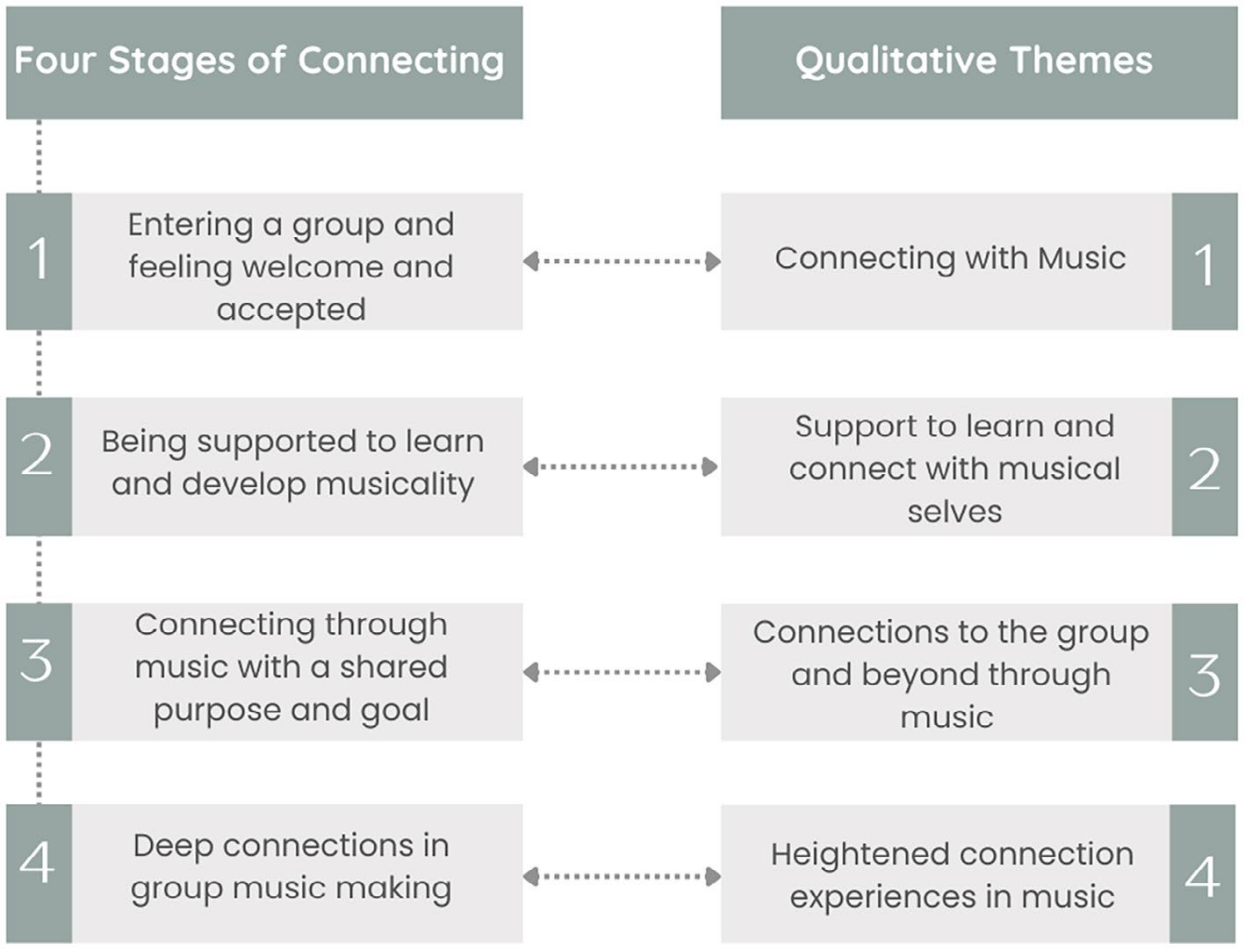
Equivalence of stages and themes.

Transcripts rendered multiple themes; however, the following were emphasised and returned to by participants from all groups: connecting with music; support to learn and connect with musical selves; connections to the group and beyond through music; and heightened connection experiences in the music. The two authors hypothesised that the four themes align with four stages of connecting, which we developed from reflections on the data and current scholarship.^13^, ^25^, ^30^, ^31^ The stages are as follows: one, entering a group and feeling welcome and accepted; two, being supported to learn and develop musicality; three, connecting through music with a shared purpose and goal; four, deep connections in group music‐making (see Figure 1 for stages and themes).

### 2.4 Connecting with music – joining

The first step to connections through music is to join a group. At this point, the accessibility of the group and the welcome a new member receives are critical. Four of the groups discussed accessibility and three discussed the welcome specifically. Three members of the four‐piece band (guitars, mandolin, and voice [*The fourth member was overseas at the time of discussions*]), specified the welcome as the first requirement of a group, which was also noted by Heidi from the women's choir, ‘I've always loved, from the instant I started, how welcome everybody is’. One member of the string band shared the acceptance that keeps him involved, ‘I love people's tolerance of my attempts at playing music’ (Quentin). Members of both choirs stressed the importance of accessibility through no requirement to read music or audition. Union choir member, Angela, stated, ‘all you have to do is turn up on a Monday’, and another member, Babs explained, ‘It's just a matter of whoever is interested enough to be a part of it’.

For the women's choir, the underlying principle is that everyone can sing, and everyone is valued, which confers acceptance, ‘And there were a couple of people in the, in the group … who really were, you know, off note. And we just encouraged them to keep singing, because everybody can’ (Dawn). The welcome and acceptance are linked to belonging and being recognised.^32^ This contrasts with the feeling of becoming invisible as Heidi from the women's choir shared:

Talking about the invisibility of the 70 and 80‐year‐old people, how you feel nobody's looking at you anymore, or taking notice of you … But when you're, like, in a choir, you're part of something, you're seen, you're a voice.

This was endorsed by Mark, who had a negative experience in a prior group, where he felt unnoticed and unvalued; he subsequently joined the string band and recounted, ‘the feeling I had was that I was welcomed there, and they wanted me there’.

### 2.5 Support to learn

Learning and personal growth are important, but not always accessible, to older adults.^1^ Some interviewees expressed gratitude at being accepted as they were and at their musical level. This was crucial for those who lacked confidence. As a new member of the string band, Will stated, ‘I like learning … everyone's very patient. Yeah, I'm just finding it really good’. Acceptance is a key aspect of feeling supported, as a choir member stated, ‘I think it's that unconditional acceptance of everyone that just makes it work’ (Lisa). Acceptance also requires a non‐judgmental music space for learning and performing, sometimes this is an aspect of the group's sound as Noreen from the string band described, ‘we can lose ourselves [in] that big wall sound, so there's no standards, there's no entry requirement’.

The facilitator is key to modelling and encouraging peer support. This support is essential for some members to progress and overcome negative self‐perceptions. One member of the women's choir talked about being given permission to sing, ‘I think it's a lot to do with that permission, to actually express yourself through singing’ (Doreen). She went on to explain how the group supported to overcome internal barriers, ‘And it's lovely being in a group that carries you through all of that, to bring you to a point where you can sing out’.

Being supported enabled participants who lacked self‐belief to realise their musical selves, whether described as being given permission to sing (Doreen); ‘to be able to explore singing and feel as though you didn't have to be perfect’ (Angela); or simply learning guitar through the patience of the group (Will). Fourteen participants stated they had no opportunities to sing in a choir or learn an instrument growing up. Three participants from the union choir were deeply affected when discouraged from singing at school, as Beatrice recounted, ‘And the teacher would go along in front of us while we were singing, and she marked me out … I took decades to recover from that rejection’.

The journey of musical discovery was described by a member of the ukulele group as moving from playing ‘a string and then a chord’ (Deirdre), to graduating to playing and singing songs. She added, ‘I love music, but I hadn't played an instrument before … It's very challenging but just, everyone is so supportive’. Another ukulele group member appreciated the group's positive and supportive learning environment, which (in contrast to an earlier experience) meant that ‘I've been learning more about how to play the ukulele musically’ (Eddie), and Georgia from the same group expressed it as the support for, ‘keeping on learning rather than getting stuck’.

Warwick from the union choir shared how the support to develop as a songwriter was ‘something that the choir has given me, that I never would've got any other way’. Interviewees also noted the access to knowledge, and social and musical worlds the groups afford. Aaron shared that joining a choir was ‘just opening up a whole new world of music’. In the ukulele group, there were two members who had tried to teach themselves to play guitar unsuccessfully, ‘I had this long career of no success and no improvement’ (Faith) and therefore appreciated the support to be successful with ukulele the group offered. Other members who had access to piano lessons or choir membership as children were grateful for the opportunity to develop musically and to find this new purpose in life. As a member of the four‐piece band shared, ‘music is my life now’ (Mark).

### 2.6 Connections to the group and beyond through music

The experience of connecting to each other was described in diverse, meaningful ways; a member of the ukulele group, Yvonne, described people's reactions to getting on well with each other, ‘they're so surprised that they get on with the whole group. And so, I feel like there must be something about it’. She considered it must be related to the joy of playing, ‘You're singing, and you're playing, and that's what is the joyful bit of it’. Then, she reflected further on what happens when they play together, ‘we're all interacting with everybody, and there's smiles on everybody's faces … it must flow over into our social interactions, you know, when we stop and have coffee’.

Connections through music can also build empathy beyond the music group. Some groups have special connections through beliefs and causes. For example, the union choir is passionate about social justice, which may explain their long history, ‘I think it's because we've got that political thread, political cause, that sort of holds it together’ (Warwick). The women's choir draws together to support women refugees with whom their facilitator works, ‘the choir came on board going, well what can we do?’ (Edith). Two facilitators of the string band play in local aged‐care facilities; and the four‐piece band plays at a dementia café.

Participants were enthusiastic about the experience of working together as a group towards learning or performing a piece of music. One interviewee emphasised the sense of working together and being in that musical space, ‘When you sit with people and you can hear the harmony and the product of what you're doing … it's a good feeling’ (Warwick, union choir). In contrast, as a member of the ukulele group shared, performing together could be negative when preparation was lacking, ‘sometimes it just turned into a mad scrambly thing where people were not playing very well’ (Ian).

### 2.7 Heightened connections and experiences in the music

Some participants also shared their experience of being together in the music and communicating non‐verbally. This experience was partly about sharing emotions elicited by the music, for example the ‘joyful bit’ (Yvonne). It is also about the non‐verbal communication, which interviewees tried to articulate in terms of feeling strongly connected and listening, ‘there's blending and there's acceptance; just builds into that thing of being there and realising that you're not – it doesn't – you're a part of something sounding really amazing’ (Frances, women's choir). In a discussion, members of the string band shared the togetherness that unified sound and rhythmic patterns could engender, articulated by Val, ‘we're all playing the same thing, and we've got about four basic patterns that we play and that's what brings us together’.

With experience, and the development of musical skills and critical listening, there was the possibility to become more tuned into the music as a whole (rather than focussing on an individual part). Participants could appreciate the combined sound when well prepared, as Sam from the ukulele group explained, ‘I feel that if you have practiced the song enough, you can have the ability to kind of pull your attention back a little bit and listen to the sum of the players and singers’. Members of the four‐piece band also described the extra‐special heightened feeling when a performance went very well. ‘I find, especially with this group … I just get the pins and needles, and you just think, oh, it just sounds so fabulous’ (Noreen). And Oswald, another group member added, ‘When I'm playing … when we really get it right, I say, “Wow, how good was that?” And I was just speechless’.

## 3 DISCUSSION

Community music affords opportunities to make new connections and find new purpose in later life.^16^ However, many would‐be music‐makers lack confidence, and therefore, the welcome they receive within a non‐judgmental learning environment is vital.^5^ Higgins^30^ advocates for community music as a potential space of generosity and welcome, while highlighting the potential of the welcome to be conditional because of groups' goals. The women's choir is envisioned as a space you can inhabit as you are, a place of connections, refuge or self‐affirmation.^33^ The choir's focus on connections and spontaneity, would as Frances explains, be adversely affected if the choir was strongly focussed on performing ‘especially if it's around getting things to sound really perfect’. Older music participants also wanted to be recognised in the sense of what they brought to the music group, ‘to be visible’ (Heidi). Dabback and Smith^34^ discuss the importance of acknowledging the rich knowledge, experience and motivation of older adults in the adult learning environment. Ernst^35^ highlights the lifetime of musical memories that each older adult possesses, alongside attributes such as work ethic and a positive attitude, which can facilitate growth and learning.

Although not the focus of this article, a key element in any community music group is a facilitator who models acceptance and encouragement, and sees participants ‘as individuals with the potential to grow in musical expertise’, as argued by Varvarigou et al.^13^ ^(p. 194)^ Interviewees talked about being supported to learn, which largely referred to the emotional support from others, expressed in terms of feeling good, and the joy of sharing music.^19^ These emotions and acceptance contribute to a safe and positive space, one that fosters trust, openness and cooperation, as outlined by Knowles et al.^36^ A generosity, described by Varvarigou et al.,^13^ was evident from members in relation to support that is voiced in comments such as feeling carried by the group to the point of singing out (Doreen); of the patience of peers by Will; of the gift of song‐writing from the choir (Warwick); and support to keep going rather than get stuck (Georgia). Such support enables the development of musical selves. These ‘possible selves’ are especially significant for older adults who had little to no early music experiences, including those who had negative (even traumatic) early music experiences.^21^ Finding and developing musical selves comes through strongly in participants' expressions, such as ‘music is my life now’ (Mark). Doing this with others and feeling their emotional support is important in particular for those who are exploring a previously denied musical self.^17^ Accessing such ‘impossible selves’ may have deep significance and meaning for some, shown by interviewees' expressions of gratitude.^37^, ^38^

Music groups are also communities, as the interviewee responses show. In an article on community music practice, Schiavio et al.^25^ discuss three themes: mutual collaboration; non‐verbal communication; and a sense of togetherness. Mutual collaboration is strongly represented in these groups, both within their music‐making and beyond. Members are active in their group's organisation, in collective activities outside rehearsing, such as song‐writing, as well as having input into repertoire and values of the group, ‘like we're all collaborating, actually very disciplined’ (Bronwin, women's choir).

The strong sense of togetherness and bonding grows through the shared musical space of interaction, which calls on deep listening, playing and responding.^25^ When playing with others we anticipate and constantly adjust to fit with musical lines, tempos and expressive techniques such as *rubato*.^25^ This was referred to by Frances as ‘there's blending and there's acceptance’ and is associated with deeper connections. It is an in‐the‐moment focus, which is linked to a flow experience but also a feeling of being in the music with others working together.^25^ Music is primarily an emotional medium and being in the moment together, sharing and communicating emotions, is another powerful experience expressed by Yvonne as the shared ‘joyful bit’ and by Val as the ‘patterns we play … [that] bring us together’.^39^ Communication while rehearsing is mainly non‐verbal, calling on multi‐sensory awareness and what Schiavio et al.^25^ ^(p. 713)^ describe as ‘in‐the‐moment interactivity’. The powerful experience of being immersed in the music as a player and connected to others creating the music with you can prompt the heightened experiences interviewees referred to as the feeling when it's ‘just right’ (Oswald, four‐piece band), or the ability to step outside yourself and hear ‘the sum of the players and singers’ moving ahead (Sam, ukulele group).^8^

### 3.1 Limitations

Those who join a music group later in life choose to do so, and if disappointed with a group, can and do move on to another. A limitation of this case study, therefore, is that disgruntled and disappointed voices are missing, although some expressed disappointment with a previous group. Another limitation is that the impact of COVID‐19 was still being felt, meaning numbers were down in all groups. In terms of ethnic representation, participants were representative of Hobart's older demographic, of which English, Australian, Irish, Scottish and First Nations are the prevalent ancestries.^40^

## 4 CONCLUSIONS

In this case study, older adult members of music groups shared some of their aspirations and positive musical experiences, including developing musical skills; realising musical selves; and feeling a deep sense of belonging. They emphasised the key roles that social support and a safe learning environment played in making space for such positive, even transformative experiences. In particular, they noted the importance of the welcome; feeling accepted; there being no judgement; being able to be themselves; and the enabling of social connections. In terms of transformation, group music‐making can lead to feelings of deep connection to others and the music, opening possibilities of empathy for others; heightened experiences; and musical awareness. Future research might focus further on the enablers to joining a music group and developing musical selves through further close work with music group participants.

## FUNDING INFORMATION

Helen English acknowledges the funding support of the Australian Research Council for this project.

## CONFLICT OF INTEREST STATEMENT

No conflicts of interest declared.

## Supporting information


Appendix S1

## Data Availability

The data that support the findings of this study are available on request from the corresponding author. The data are not publicly available due to privacy or ethical restrictions.
