# Supplementary material for: Music groups and connectivity: Older adults' perceptions of socialising through community music
Source: Australas J Ageing. 2025 Jun 22;44(2):e70057. doi: 10.1111/ajag.70057 (PMC12183150; doi:10.1111/ajag.70057)
Supplement: Supplementary file 1 — Appendix S1 [file AJAG-44-0-s001.docx]

**Appendix 1**

Interview questions for creative ageing project 2023-2024: These are starting points only.

1. Tell me something about your early musical experiences
2. Did you continue with music in adulthood?
3. When did you join this group? How did you hear about it?
4. What keeps you coming every week?
5. Do you feel it challenges or extends you?
6. Do you enjoy the repertoire? Do members have input into this?
7. Tell us about the facilitator
8. Does your group perform? If yes, is this important for you?
9. What does music do for you? (thinking about benefits, purpose, new skills/growth etc)
10. How important is music in your life?
11. Has being a member of this music group changed anything for you? (might be new social circle, new musical identity, new purpose, new way of listening, new interest to diverse genres; new confidence etc)
